# Phytochemistry and Pharmacological Potential of the Mangrove Plant *Sonneratia caseolaris*: A Comprehensive Review

**DOI:** 10.3390/md23100378

**Published:** 2025-09-26

**Authors:** Federico Cerri, Paolo Galli

**Affiliations:** 1 Department of Earth and Environmental Sciences DISAT, University of Milano-Bicocca, Piazza della Scienza 1, 20126 Milan, Italy; paolo.galli@unimib.it; 2 MaRHE Centre (Marine Research and Higher Education Center), Magoodhoo Island, Faafu Atoll 12030, Maldives

**Keywords:** mangroves, *Sonneratia caseolaris*, traditional medicine, natural products, bioactive compounds, phytochemical analysis, pharmacological activities, antioxidant activity, antimicrobial activity, anticancer potential

## Abstract

Mangroves represent a promising yet underexplored source of natural products. *Sonneratia caseolaris* (mangrove apple) is a widely distributed species with a long history of use in traditional medicine, and it is receiving increasing recognition for its bioactive secondary metabolites. Research has expanded in recent decades, but findings remain dispersed across diverse sources, complicating interpretation of its chemistry and pharmacological potential. This review consolidates four decades of investigations, documenting 141 identified compounds from studies largely restricted to India, Bangladesh, Indonesia, and China and focusing on leaves, fruits, bark, stems, and twigs, with roots notably unexplored. The phytochemical profile is dominated by phenolic acids, flavonoids, and tannins, alongside terpenoids, steroids, fatty acids, fatty alcohols, aldehydes, hydrocarbons, and polysaccharides. The most extensively studied activities are antioxidant and antimicrobial, with extracts consistently exhibiting strong free-radical scavenging capacity and broad-spectrum antibacterial and antifungal effects, including efficacy against drug-resistant strains. Additional reports describe central nervous system depressant, antidiarrheal, metabolic, anti-inflammatory, analgesic, antipyretic, and anti-allergic activities. In contrast, anticancer investigations remain scarce, despite promising outcomes reported for related mangrove taxa. By consolidating and critically evaluating the existing evidence, this review highlights the pharmacological potential of *S. caseolaris* and identifies key knowledge gaps to guide future marine drug discovery.

## 1. Introduction

Mangroves are among the most biologically, ecologically, and economically important ecosystems in the world. In addition to their well-known ecosystem services, such as coastal protection, carbon sequestration and food provision [1,2,3,4], mangroves also serve as a source of compound for drug discovery [5]. For decades, plants have been extensively studied for drug discovery [6], and mangroves are particularly promising for several reasons. Their unique genetic diversity, the biodiversity of their ecosystems, where complex interactions between biotic and abiotic factors influence the production of a wide array of molecules, their ability to thrive in extreme environments that triggers the synthesis of specialized metabolites as adaptive strategies, and their long-standing use in traditional medicine all contribute to their pharmaceutical potential [7,8,9]. However, compared to many other plants, research on mangrove species remains relatively recent and limited [10,11].

**Figure 1:**
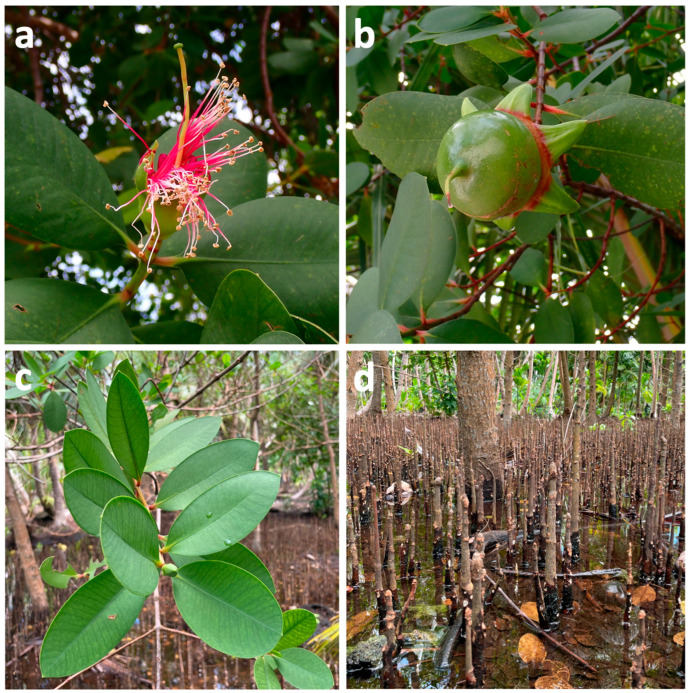
Flower (**a**), fruit (**b**), leaves (**c**), and roots (**d**) of *Sonneratia caseolaris*.

Among mangrove genera, *Sonneratia* which belongs to the Sonneratiacea family [12], has attracted significant attention in this field [13]. In particular, *Sonneratia caseolaris* (L.) Engl. has been of interest as it has been widely used in folk medicine to treat ailments such as sprains, piles, cuts and bruises, hemorrhages, intestinal parasites, diarrhea, coughs, hematuria, smallpox, and hepatitis [14,15,16] and is known to produce a wide range of polyphenols with various biological activities [13,17,18,19]. Commonly known as mangrove apple [4], *S. caseolaris* is a mangrove tree that can grow up to 20 m in height. It is characterized by light green, rounded, leathery fruits up to 8 cm in diameter, red-petalled flowers, elliptic coriaceous leaves, and long, pointed pneumatophores [16,20,21] (Figure 1). This species is distributed across various regions including the Maldives, Sri Lanka, China, Bangladesh, the Malay Peninsula, Indonesia, Borneo, the Philippines, Timor, New Guinea, the Solomon Islands, and northern Australia [4,16,21,22,23].

Despite its traditional and pharmacological relevance, *S. caseolaris* has received far less systematic attention compared with other mangrove specie. For instance, Bibi et al. [24] reported 27 mangrove species validated for pharmacological activity yet *S. caseolaris* was not included. Similarly, other reviews on the anticancer potential of mangrove-derived phytochemicals [25] and on phytochemistry and bioactivities of mangrove plants [10] did not report this species. These omissions underline the significance and prospects of studying *S. caseolaris*, a promising but historically underexplored mangrove species.

In a context where specific mangrove plants are now being extensively reviewed, including *Avicennia* spp. [26,27] and *Aegiceras corniculatum* [28], the phytochemical and pharmacological data available for *S. caseolaris* remain limited and highly scattered across different sources. This comprehensive review therefore aims to systematically consolidate four decades of research on *S. caseolaris*, with particular emphasis on its bioactive compounds and pharmacological activities. The objectives are to systematically analyze all available studies, highlight the most findings, and identify key research gaps that warrant further investigation. By clarifying what is known and where uncertainties persist, this review, which to our knowledge, is the first systematic consolidation of four decades of research on *S. caseolaris* phytochemistry and pharmacological activities, provides a timely and comprehensive foundation for advancing the exploration of *S. caserolaris* as a promising candidate for drug discovery and therapeutic development.

## 2. Components of Sonneratia caseolaris

The chemical composition of *S. caseolaris* has been extensively investigated, revealing a diverse array of secondary metabolites. These include phenolic compounds, flavonoids, tannins, terpenoids, steroids, fatty acids, alcohols and aldehydes, hydrocarbons, polysaccharides, and various other constituents. Table 1 provides a comprehensive overview of the chemical composition of *S. caseolaris*, including the plant part in which each molecule was identified, the solvent used for extraction, the identification method, the region of sample collection, and the corresponding reference. Regarding the extraction methods, the majority of studies employed maceration, in which plant material, typically dried and powdered (with the exception of Tiwari et al. [29], who explicitly reported using fresh material), was soaked in solvent at room temperature for varying durations, generally 24 h or longer. One exception is Jha et al. [30], who used hot water extraction.

### 2.1. Phenolic Compounds

Phenolic compounds are abundant in *S. caseolaris*, with their concentration varying significantly depending on the plant part and extraction solvent used, as reflected by the total phenolic content (TPC) values reported in multiple studies (see Table 2). Methanol end ethanol extractions generally yield the highest phenolic contents, consistent with the polarity-dependent solubility of these compounds. Leaves consistently exhibit higher phenolic content than fruits and bark. For example, ethanol extracts of leaves typically range from 50.03 to 219.53 mg GAE/g [8,40,41], compared to 12.21–122 mg GAE/g in fruits [18,42] and 50.70–63.00 mg GAE/g in bark [36,43]. Notably, the ethanolic leaf extracts of *S. caseolaris* contain higher TPC than those of other mangrove species such as *Avicennia marina*, *Rhizophora mucronata* and *Rhizophora apiculata* [8]. Using the classification by Audah et al. [8], where the phenolic content is categorized as high (>70 mg GAE/g), moderate (10–70 mg GAE/g), and low (<10 mg GAE/g), most methanol and ethanol extracts from *S. caseolaris* leaves and fruits fall within the high phenolic concentration category. However, notable exceptions exist, such as the very low TPC (<2 mg GAE/g) reported by Kartikaningsih et al. [37] for methanol and water extracts, illustrating the significant impact of plant material, solvent composition and extraction protocols on phenolic recovery.

#### 2.1.1. Phenolic Acids and Derivatives

**Figure 2:**
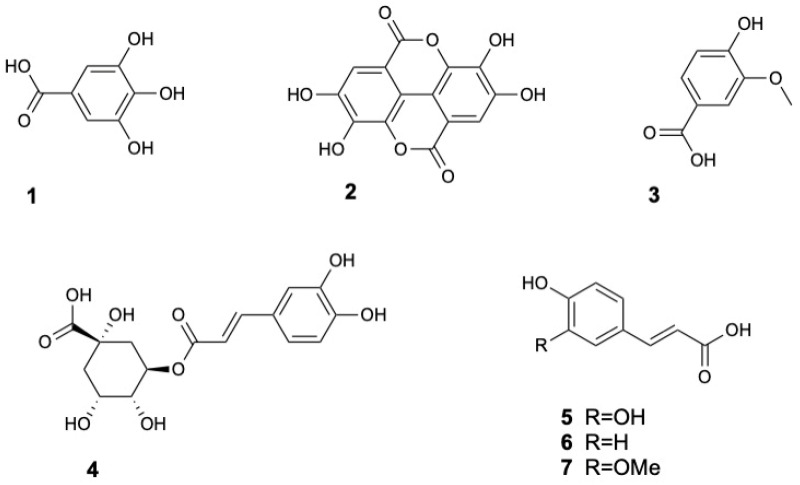
Phenolic Acids and Derivatives from *Sonneratia caseolaris*.

Phenolic acids are a major class of phenolic compounds broadly divided into hydroxycinnamic and hydroxybenzoic acid groups [44]. Hydroxybenzoic acids such as gallic acid (**1**), ellagic acid (**2**) and vanillic acid (**3**) have been identified in *S. caseolaris* leaves and fruits [8,13,16,31,32]. Hydroxycinnamic acids including chlorogenic acid (**4**), caffeic acid (**5**), p-coumaric acid (**6**), and ferulic acid (**7**) were also found in the leaves [33]. The structures of phenolic acids and derivatives **1–7** are displayed in Figure 2.

#### 2.1.2. Flavonoids

As shown in Table 2, total flavonoid content (TFC) is also high and varies with plant part and extraction solvent. Methanol end ethanol extractions typically yield the highest TFC across all tissues, though values vary widely among studies. For instance, leaf extracts range from 22.70 to 454.88 mg QE/g [8,40], fruit extracts from 26.06 to 613 mg GAE/g [18,42] and bark extracts around 90.04 ± 3.57 [36]. Importantly, *S. caseolaris* leaves consistently show higher TFC than leaves of other mangrove species such as *A. marina*, *R. mucronata* and *R. apiculata* when extracted with ethanol [8].

**Figure 3:**
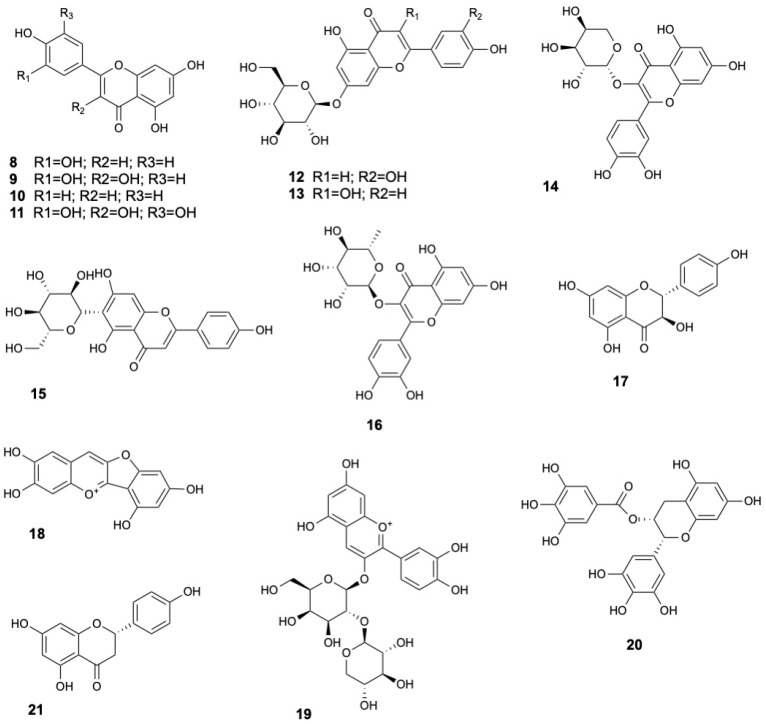
Flavonoids from *Sonneratia caseolaris*.

Several flavonoid aglycones have been consistently identified in the leaves and fruits of *S. caseolaris*, including luteolin (**8**), quercetin (**9**), apigenin (**10**) and myricetin (**11**) [13,16,21,29,31,33,34]. Flavonoid glycosides such as luteolin-7-*O*-glucoside (**12**) [13,21,34,35], kaempferol glucoside (**13**) [36], quercetin-3-*O*-β-L-arabinopyranoside (**14**) [22], isovitexin (**15**) and quercitrin (**16**) [8] have also been detected. Flavonoid (+)-dihydrokaempferol (**17**) were found in stems and twigs [22], while anthocyanin derivatives such as riccionidin A (**18**) and cyanidin 3-*O*-[β-D-xylosyl-(1-2)-β-D-galactoside] (**19**) were identified in leaves [8]. Catechins, a subclass of flavan-3-ols with well-known antioxidant properties [45], were also found, including epigallocatechin gallate (**20**) [33]. Moreover, the flavanone naringenin (**21**) was detected [33], further enriching the flavonoid diversity of the species. The structures of flavonoids **8–21** are displayed in Figure 3.

#### 2.1.3. Tannins

**Figure 4:**
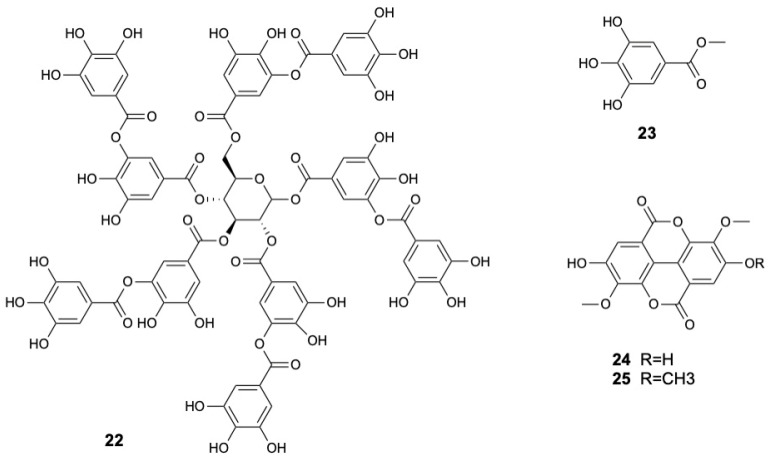
Tannins from *Sonneratia caseolaris*.

Tannins, a significant subclass of polyphenols known for their ability to complex with proteins, cellulose, and minerals, are broadly divided into hydrolysable and condensed forms. In *S. caseolaris*, the bark is a particularly rich source, with tannin content reported at 48.04 ± 0.91 mg TAE/g [36]. Fruits also contribute significantly, with the ethanol extracts yielding 30 mg GAE/g [18]. Chemical analyses have identified hydrolysable tannins like tannic acid (**22**), methyl gallate (**23**) and two ellagic acid derivatives, namely 3,3′-di-*O*-methyl ether ellagic acid (**24**) and 3,3′,4-*O*-tri-*O*-methyl ether ellagic acid (**25**) [22,31]. The structures of tannins **22–25** are displayed in Figure 4.

#### 2.1.4. Other Phenolic Compounds

Besides phenolic acids, flavonoids, and tannins, *S. caseolaris* was also found to contain various other phenolic compounds (**26–38**) extracted from its fruits, leaves, bark, and wood. These include simple phenolics, phenolic aldehydes, benzopyrans, lignans, and glycosylated derivatives. Their structural diversity highlights the rich and complex phenolic profile of *S. caseolaris* beyond the major phenolic classes [8,15,31,34,37].

### 2.2. Terpenoids and Steroids

**Figure 5:**
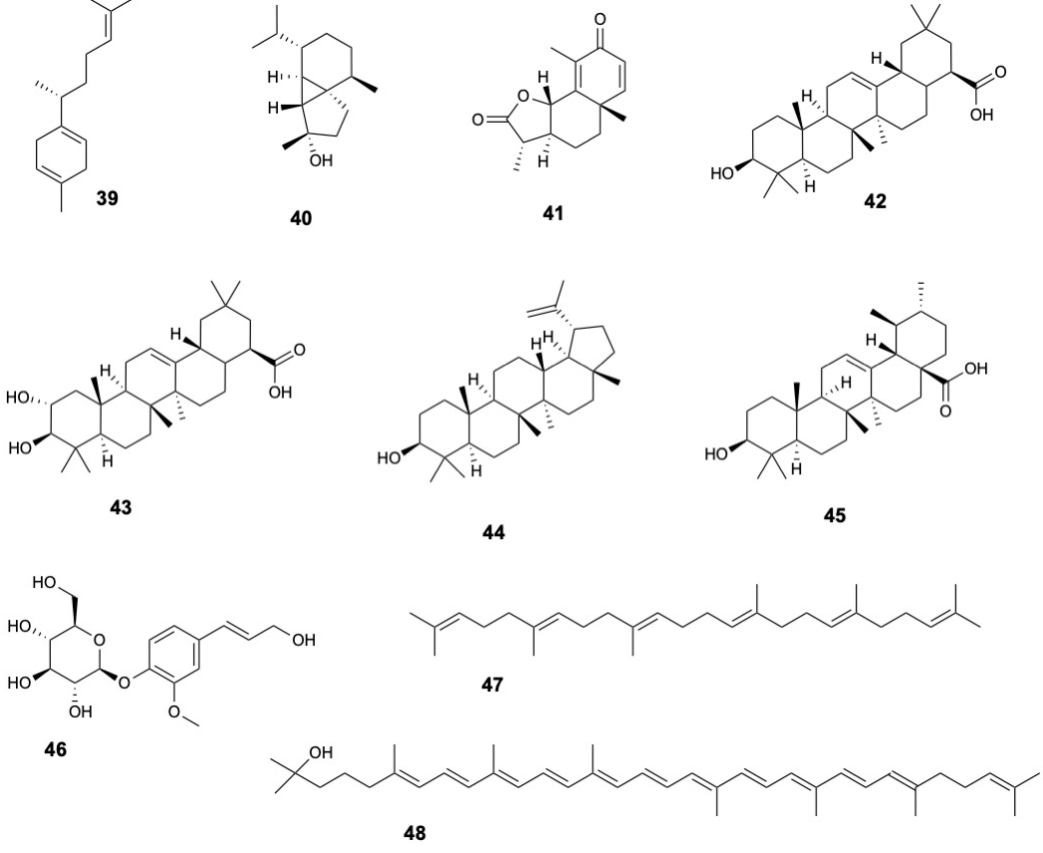
Terpenoids from *Sonneratia caseolaris*.

*S. caseolaris* contains a wide range of terpenoids. Sesquiterpenes such as β-curcumene (**39**), cubebol (**40**), and α-santonin (**41**) have been identified in fruits [19]. Triterpenoids are particularly prominent, including oleanolic acid (**42**) detected in fruits, stems, twigs, and bark [22,29,34,36], maslinic acid (**43**) in fruits [29,34], and lupeol (**44**) and ursolic acid (**45**) in stems, twigs, and bark [22,36]. Diterpenoids, such as abietin (**46**), have been found in leaves [8]. Other terpenoids identified include squalene (**47**) in leaves [38] and rhodopin (**48**) in fruits [19] and other compounds (**49**–**55**) in stems and twigs [22]. The structures of terpenoids **39–48** are displayed in Figure 5.

**Figure 6:**
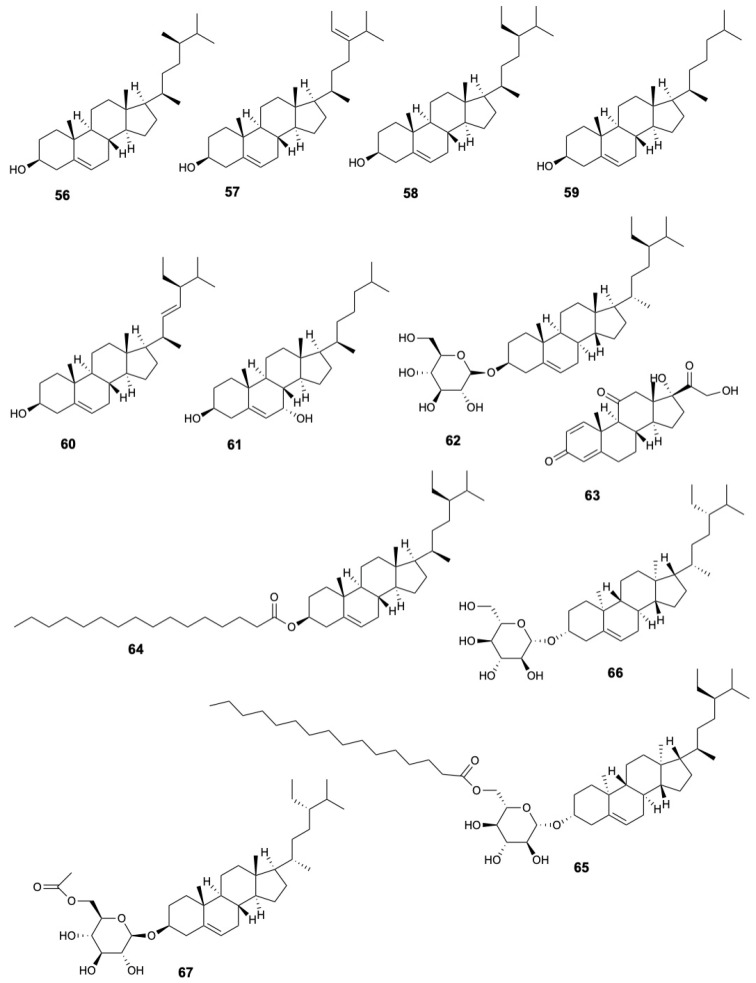
Steroids from *Sonneratia caseolaris*.

Phytosterols present in the leaves and bark include campesterol (**56**), 28-isofucosterol (**57**), sitosterol (**58**), cholesterol (**59**), stigmasterol (**60**) [22,38]. Additionally, cholest-5-ene-diol (**61**) was found in bark, stems and twigs [22,36], and the sterol glycoside β-sistosterol-β-D-glucopyranoside (**62**) in fruits [29]. The steroid prednisone (**63**) was identified in fruits [19], while stems and twigs contain various steroids and sterol derivatives including β-sitosterol palmitate (**64**), stigmast-5-en-3β-*O*-(6-O-hexadecanoyl-β-D-glucopyranoside) (**65**), and daucosterol (**66**), 6′-*O*-acetyl-β-daucosterol (**67**) [22]. The structures of steroids **56–67** are displayed in Figure 6.

### 2.3. Fatty Acids and Derivatives, Fatty Alcohols, and Fatty Aldehydes

*S. caseolaris* contains several notable long-chain fatty acids, such as 13S-hydroxyoctadecadienoic acid (**68**), 9-hydroperoxy-11-(3-pentyl-2-oxiranyl)-10-undecenoate (**69**), 9,12,13-trihydroxy-10-octadecenoate (**70**) [8]. Other fatty acids include octanoic acid (**71**) and butanoic acid (**72**), as well as derivatives like dodecanamide (**73**), and myristynoyl pantetheine (**74**) [15,19].

Fatty aldehydes identified include 2-heptenal (**75**), 2-octenal (**76**), nonanal (**77**), 2,4-decadienal (**78**), 2-undecenal (**79**), hexadecanal (**80**), tetradecanal (**81**), octadecanal (**82**), 2-nonenal (**83**), decanal (**84**) [15]. Additionally, tridecanedial (**85**) was detected in leaves [19].

Fatty alcohols reported in the leaves include 13-heptadecyn-1-ol (**86**), 2-hexadecanol (**87**), 1-octanol (**88**), and falcarinol (**89**) [19], while trans-9-hexadecen-1-ol (**90**) was found in the wood [15].

**Figure 7:**
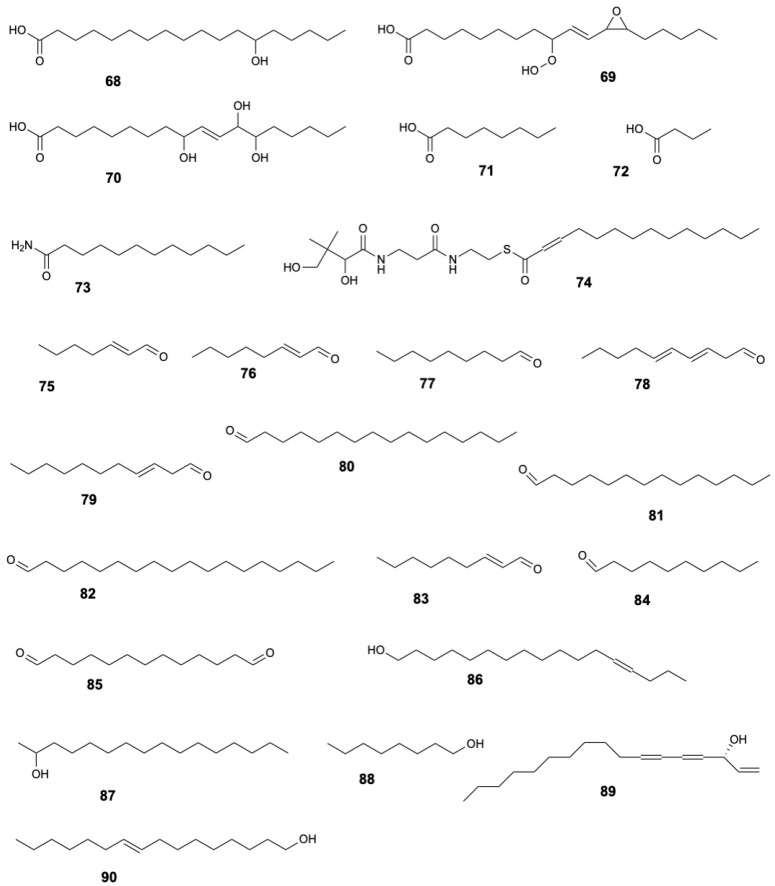
Fatty acids and derivatives, fatty alcohols, and fatty aldehydes from *Sonneratia caseolaris*.

Furthermore, other lipid-derived compounds (**91–96**) were identified in wood and leaves of *S. caseolaris* [8,15,19]. The structures of fatty acids and derivatives, fatty alcohols, and fatty aldehydes **68–90** are displayed in Figure 7.

### 2.4. Hydrocarbons, Polysaccharides, and Other Constituents

Several hydrocarbons have been detected in wood and bark extracts, including pentadecane (**97**), 1-hexadecene (**98**), 1-docosene (**99**), octacosane (**100**), and hentriacontane (**101**). Wood also contains heptadecane (**102**), 2-methyl-nonadecane (**103**), octadecane (**104**), tetracosane (**105**), and heptacosane (**106**), while bark includes eicosane (**107**), 17-pentatriacontene (**108**), isobutane (**109**) and 3-methyl-exane (**110**), and the halogenated hydrocarbon 1-chloro-heptacosane (**111**) [15].

Leaves of *S. caseolaris* are a source of carbohydrates, both simple and polymeric. Ethanol leaf extracts contain the free sugar hexose (**112**) and the sugar alcohol sorbitol (**113**) [8]. Low-molecular-weight leaf polysaccharide fraction were found to comprise rhamnose (**114**) (28.25%), xylose (**115**) (27.17%), mannose (**116**) (18.90%), and galactose (**117**) (17.17%), indicated branched heteropolysaccharide structures [30].

Kartikaningsih et al. [37] reported the presence of anthropogenic contaminants in leaves, such as diisobutyl phthalate (**118**), bis(3,5,5 trimethylhexyl)phthalate (**119**), monobutyl phthalate (**120**), and bis(2 ethylhexyl)phthalate (**121**), along with naturally occurring nitrogenous and zwitterionic compounds including betaine (**122**), choline (**123**), hexamethylenetetramine (**124**), and the nitroxide 2,2,6,6 tetramethyl 1 piperidinol (TEMPO) (**125**). Additional constituents include caprolactam (**126**), 2-[(2-chlorobenzyl)sulfanyl]-4,6-dimethylnicotinonitrile (**127**), the mycotoxin zearalenone (**128**), tributyl phosphate (**129**), bis(4-ethylbenzylidene)sorbitol (**130**), and the amino acid DL-arginine (**131**). Stems and twigs yielded benzenecarboxylate derivatives such as bis(2-ethylhexyl)benzene-1,2-dicarboxylate (**132**) [22], while fruits contain safrole (**133**) [19]. Furthermore, Wood and bark contained phthalate esters, spiroketones, and amines (**134–141**) [15].

## 3. Biological Activities

### 3.1. Safety and General Toxicity

#### 3.1.1. Acute and Subacute Toxicological Screening

Dev et al. [16] conducted both acute and sub-acute toxicity studies on male Swiss albino mice using the fruit ethanol extract of *S. caseolaris*. In the acute assay, doses up to 3000 mg/kg body weight produced no mortality or behavioral abnormalities, indicating a high safety margin. For the sub-acute test, mice received 500 mg/kg daily for 14 days; there were no significant changes in biochemical markers (urea, creatinine, bilirubin, SGPT, SGOT), confirming the extract’s safety as a crude herbal medicine. These findings align with Kundu et al. [46], who observed no mortality in Swiss albino mice treated acutely with up to 5000 mg/kg of the same extract over 7 days.

#### 3.1.2. Brine Shrimp Lethality Assay

Shamsuddin et al. [47] assessed the toxicity of aqueous and ethanolic leaf extracts via *Artemia salina* lethality. The LC_50_ values were 0.8 µg/mL and 6.3 µg/mL, respectively, suggesting potential cytotoxic constituents. Bokshi et al. [48] fractionated ethanol extracts of leaves and stems, identifying the ethyl acetate stem and CCl_4_ leaf fractions as most toxic with LC50 values of 25.0 ± 0.07 and 25.0 ± 0.05 µg/mL, respectively, compared to vincristine sulfate (LC_50_ of 0.156 ± 0.09 µg/mL). Hosen et al. [49] reported weak lethality for ethanol:methanol (1:1) fruit extract with a LC_50_ value of 444.3 µg/mL (vincristine sulphate: 0.45 µg/mL) while Kundu et al. [46] found the fruit ethanol extract LC_50_ of 219.3 µg/mL (vincristine sulphate: 0.584 µg/mL).

### 3.2. Antimicrobial and Antiparasitic Activities

#### 3.2.1. Antibacterial and Antifungal Activity

*S. caseolaris* demonstrated broad-spectrum antimicrobial properties, with activity reported against a wide range of Gram-positive and Gram-negative bacteria, drug resistant strains, and fungal pathogens (see Table 3 for full dataset). The antimicrobial effects vary markedly by plant part, solvent, and target organism. All available studies of antibacterial and antifungal activities of *S. caseolaris* extracts have been conducted in vitro, with no in vivo or clinical evaluations yet available.

Leaves have been the most extensively investigated and exhibit consistent antibacterial and antifungal activity. Yompakdee et al. [50] reported minimum inhibitory concentration (MIC) values ranging from 0.2 to 0.4 mg/mL against antibiotic-sensitive and resistant strains, including methicillin-resistant *Staphylococcus aureus* (MRSA). The activity was corroborated by Audah et al. [8], who confirmed inhibition of MRSA, and Nguyen et al. [13], who observed potent inhibition of *S. aureus*, *Eschirichia coli*, and *Salmonella typhimurium*, with inhibition zones (ZOIs) exceeding 25 mm and MICs as low as 0.04 mg/mL. Additionally, Shamsuddin et al. [47] found that methanol leaf extract inhibited six *Vibrio* species (12–18.3 mm). Beyond direct antimicrobial effects, Jha et al. [30] demonstrated antibiofilm activity of a low-molecular-weight polysaccharide fraction against *Pseudomonas aeruginosa*, *Streptococcus pneumoniae*, *S. aureus*, *E. coli*, and *S. typhi*. The antifungal activity of leaf extracts has also been well documented. Ethyl acetate extracts exhibited strong activity against *Candida albicans*, *C. tropicalis*, and *C. auris* (MIC 4–32 mg/mL) [33], while Kaewpiboon et al. [51] reported activity from water extract against *C. albicans* (ZOI 15 mm; MIC 125 µg/mL). Pagarra et al. [41] also observed inhibiyion by 70% ethanol and n-hexane extracts.

Fruits also displayed significant antimicrobial properties. Multiple studies reported inhibitory effects of crude and fractionated extracts against *Bacillus subtilis*, *E. coli*, *S. aureus*, *S. typhimurium*, and *Vibrio cholerae* [19,53,55], together with several antibiotic-sensitive and resistant strains [50]. Hosen et al. [49] found that ethanol-methanol (1:1) extracts showed inhibition zones of 14.7–17.0 against *E. coli*, *Klebsiella* sp., *Shigella boydii*, *S. sonnei*, and *S. aureus*. Fruits extracts also exhibited antifungal activity, with inhibition of *C. albicans* by methanol (7.03 mm), ethyl acetate (11.67 mm), and 70% ethanol (28.50 mm) extracts [54,55].

Other plant parts such as seeds, flowers, roots, bark, and stems have received less attention but still show promising results. Methanol seed extract inhibited *S. aureus* (12–14 mm) and *C. albicans* (17–18 mm) [52], while Yompakdee et al. [50] reported antibacterial activity from flowers, seeds, roots against both antibiotic-sensitive and resistant strains. Bark extracts, particularly methanol fractions, were found to be effective against Gram-positive species such as *B. subtilis* and *B. coagulans* [36]), though they lacked activity against *E. coli* and *S. cerevisiae*. Stem fractions, especially those derived from ethyl acetate and carbon tetrachloride, showed activity against *Salmonella* spp. [48], indicating that targeted solvent extraction may enhance antimicrobial efficacy.

#### 3.2.2. Anthelmintic Activity

Kundu et al. [46] evaluated ethanol fruit extract ex vivo on *Paramphistomum cervi*. At 12.5, 25, and 50 mg/mL, paralysis occurred in 39.6, 29.5, and 20.6 min and death in 40.3, 36.8, and 29.9 min, while the standard albendazole (15 mg/mL) induced paralysis/death in 8.6/18.5 min.

#### 3.2.3. Antiplasmodial Assay

Mulhaimin et al. [56] evaluated ex vivo anti *Plasmodium berghei* strain ANMKA activity by testing methanol leaf extract into infected Balb-C mice blood, achieving almost 60% suppression at 300 µg/mL (90% for the standard pyrimethamine at 100 µg/mL).

### 3.3. Antioxidant Activity

The antioxidant potential of *S. caseolaris* has been widely evaluated using a variety of in vitro assays, including radical scavenging activity (DPPH, ABTS, superoxide, hydrogen peroxide), reducing power (FRAP), and metal chelation methods. These approaches have consistently demonstrated notable antioxidant properties in different plant parts. To date, the available evidence is limited to in vitro evaluations, and no in vivo studies have been reported.

#### 3.3.1. DPPH Scavenging Activity

The 2,2-diphenyl-1-picrylhydrazyl (DPPH) assay has been widely applied to evaluate the free-radical scavenging capacity of *S. caseolaris* (Table 4) with leaf extracts being the most extensively studied and generally showing strong antioxidant activity.

Ethanol has emerged as the most effective solvent for leaf extract, with Pagarra et al. [41] reported up to 80.21% DPPH scavenging using 96% ethanol at 2.5 mg/mL. In a comparative screening of 53 medicinal plants, Kaewpiboon et al. [51] identified *S. caseolaris* leaf ethanol extract as the most potent, with an EC_50_ values of 1.92 ± 0.38 μg/mL, substantially lower than the positive control, ascorbic acid (EC_50_: 12 ± 1.29 μg/mL). However, other studies reported higher IC_50_ values for the same extract, ranging from 4.25 to 171 μg/mL [8,13,39,40,57], likely reflecting differences in plant origin, extraction conditions and assay protocols.

Fractionation of ethanol leaf extracts using solvents of varying polarity revealed a clear polarity-dependent trend in antioxidant activity. Bokshi et al. [48] reported IC_50_ values of 12.0 ± 0.12, 19.0 ± 0.07, 49.0 ± 0.05, for ethyl acetate, chloroform and carbon tetrachloride fractions, respectively. In contrast, stem fractions showed weaker activity, with the chloroform fraction being the most effective (IC_50_: 69.0 ± 0.21 µg/mL; AA: 8.0 ± 0.12 µg/mL). Additionally, specialized fractions of the ethanol leaf extract also demonstrated antioxidant activity: an ellagitannin-rich leaf yielded an IC_50_ of 69.39 ± 0.29 µg/mL, surpassing the butylated hydroxyanisole BHA (IC_50_:116.52 ± 0.95 µg/mL) [32], while a low-molecular-weight polysaccharide fraction achieved 41.33 ± 0.82% scavenging at 3.2 mg/mL (AA: 85.26  ±  0.96%) [30].

Fruit extracts have likewise been explored for DPPH scavenging activity. Ethanol extracts demonstrated IC_50_ values ranging from 1.16 to 87 μg/mL [18,42]. Additionally, ethanol-methanol (1:1) extract revealed a 48.1% scavenging at 50 µg/mL [58], while aqueous extract displayed an IC_50_ values of 7.3 mg/mL [53].

Bark extracts, though less frequently studied, have also demonstrated significant DPPH radical scavenging activity. Ethanol bark extract exhibited strong antioxidant activity with IC_50_ of 4.57 µg/mL, while petroleum ether, ethyl acetate, and chloroform extracts showed IC_50_ values of 12.32, 13.09, and 192.27 µg/mL, respectively (BHT: IC_50_ of 3.25 µg/mL) [43]. Additionally, the methanol bark extract exhibited an IC_50_ of 21.74 µg/mL [36].

#### 3.3.2. ABTS Scavenging Activity

ABTS•+ scavenging assay complements DPPH by measuring both hydrophilic and lipophilic antioxidants. An ellagitannin-rich leaf fraction showed strong activity with an IC_50_ of 45.11 ± 0.49 µg/mL, outperforming BHA (IC_50_ of 88.46 ± 0.11 µg/mL) [32]. Siamsul et al. [59] reported very low IC_50_ values for leaf ethanol extracts (1.53 ppm; AA: 0.72 ppm), with fractionation yielding IC_50_ values of 19.89 (n-hexane), 0.50 (ethyl acetate), and 1.63 ppm (ehtnaol). Fruit ethanol extracts recorded IC_50_ of 97.32 ± 3.27 µg/mL (AA: 12.71 ± 1.14 µg/mL) [42]. In addition, the low-molecular-weight leaf polysaccharide fraction exhibited a scavenging effect of 87.13  ±  0.66% at 3.2 mg/mL, lower than that of ascorbic acid [30].

Wetwitayaklung et al. [60] assessed ABTS•+ radical scavenging using the TEAC method and also reported IC_50_ values for various plant parts of *S. caseolaris* obtained through both maceration and Soxhlet extraction. Methanolic macerates of different organs showed IC_50_ values ranging from 13.64 to 31.95 µg/50 µL, with the calyx of flowers demonstrating the strongest activity (TEAC: 0.69). Soxhlet extraction with methanol yielded IC_50_ values between 10.38 and 51.61 µg/50 µL, with seeds exhibiting the highest antioxidant potential (TEAC: 0.96). Ethyl acetate Soxhlet extracts showed IC_50_ values ranging from 8.85 to 37.65 µg/50 µL, where the stamen displayed the greatest activity (TEAC: 1.05). In contrast, Soxhlet extraction with dichloromethane resulted in significantly higher IC_50_ values (277.75–2293.79 µg/50 µL), indicating substantially weaker antioxidant performance.

#### 3.3.3. Reducing Power and Ferric-Reducing Antioxidant Power (FRAP)

Reducing power assays measure electron-donating capacity. A fruit ethanol–methanol (1:1) extract showed a reducing power (OD) of 0.54 at 0.3 mg/mL [58]. Leaf ethanol extracts recorded RC_50_ of 395.5 µg/mL [40], while fruit ethanol extracts displayed FeCl3 reducing power with RC_50_ value of 80 µg/mL (AA RC_50_: 28 µg/mL) [18]. Siamsul et al. [59] reported leaf ferric reducing antioxidant power (FRAP) values of 345.125 ± 4.196 mM/g, highlighting strong redox activity. Wetwitayaklung et al. [60] also assessed FRAP activity using the GEAC method, expressing results as gallic acid equivalent antioxidant capacity. Extracts obtaining using methanol (maceration and Soxhlet extractions) and ethyl acetate (Soxhlet) of different plant parts exhibited GEAC values were in range of 0.06–0.23.

#### 3.3.4. Hydrogen Peroxide and Superoxide Scavenging

Fruit ethanol extract scavenged H_2_O_2_ with EC_50_ of 66 µg/mL and superoxide (O_2_•–) with EC_50_ of 347 µg/mL, while ascorbic acid exhibited EC_50_ of 11 µg/mL and 111 µg/mL, respectively [18]. Jha et al. [30] reported that low-molecular-weight polysaccharide fractions from leaves achieved 66.0 ± 1.00% superoxide scavenging at 3.2 mg/mL (AA: 95.62  ±  1.05 at 3.2 mg/mL)

#### 3.3.5. Metal-Chelation Assay

Heavy-metal chelation mitigates oxidative catalysis. Leaf polysaccharide fractions chelated of 69.25  ±  1.18% Fe^2+^ at 3.2 mg/mL, compared to EDTA-2Na (92.44  ±  0.37%) as the positive control [30].

### 3.4. Anti-Inflammatory and Analgesic Effects

#### 3.4.1. Anti-Inflammatory Activity

The anti-inflammatory activity of *S. caseolaris* has been primarily evaluated in vivo using mouse models. Kundu et al. [18] demonstrated that fruit ethanol extract (250–500 mg/kg body weight) significantly reduced formalin-induced paw edema in mice, although the effect was less than that of ibuprofen (100 mg/kg). Munira et al. [61] reported that the stem petroleum ether fraction (150–300 mg/kg) inhibited edema by 37.7% at 4 h, which was lower than indomethacin (62.3% at 10 mg/kg). Similarly, Munira et al. [62] found that leaf methanol extract (100–200 mg/kg) inhibited edema by 58.2–60.4%, compared to 94.9% inhibition by indomethacin (10 mg/kg).

#### 3.4.2. Analgesic Activity

The analgesic potential of *S. caseolaris* has been evaluated in vivo in mice, using both peripheral and central nociception models. Peripheral analgesic activity was reported by Ahmed et al. [63], where ethanol leaf extract (250–500 mg/kg b.w.) inhibited writhing by 21–48%, comparable to diclofenac sodium (40% inhibition at 25 mg/kg b.w.). Bokshi et al. [64] noted writhing inhibition of 33% and 17% at 250 mg/kg, and 50% and 54% at 500 mg/kg, for ethyl acetate stem and chloroform leaf fractions, respectively (diclofenac sodium: 65% at 25 mg/kg b.w.). Additionally, Munira et al. [61,62] found higher inhibition rates of 78.23% and 73.82% at 200 mg/kg b.w. for methanol leaf and bark extracts (diclofenac sodium: 95.58% at 10 mg/kg), and 52.45–79.40% for various stem fractions at 300 mg/kg (diclofenac sodium: 82.78% at 10 mg/kg), while Kundu et al. [18] showed fruit ethanol extract (250–500 mg/kg) inhibited writhing by 20.7–39.3%.

For central analgesic activity, Munira et al. [61] used the formalin-induced paw licking test in mice, where stem extracts inhibited nociceptive activity during both neurogenic (0–5 min) and inflammatory (15–30 min) phases. The ethyl acetate and chloroform fractions were the most effective, showing 72.91% inhibition at 300 mg/kg in the late phase (diclofenac sodium: 77.08% at 10 mg/kg). Furthermore, Kundu et al. [18], using the tail immersion method, found that fruit ethanol extract (250–500 mg/kg) increased latency by 22.5–37.5%, compared to tramadol (55.84% at 10 mg/kg).

### 3.5. Central Nervous System (CNS) Modulation

The central nervous system (CNS) modulatory potential of *S. caseolaris* has been investigated through in vivo behavioral assays to assess CNS-depressant effects, as well as in vitro enzyme inhibition studies to evaluate anticholinesterase activity.

#### 3.5.1. CNS-Depressant Activity

Munira et al. [61] assessed stem fractions (150–300 mg/kg body weight) using the hole cross test and observed moderate CNS depressant effects at 90 and 120 min, compared to diazepam (1 mg/kg). Similarly, Kundu et al. [46] reported that fruit ethanol extract (250–500 mg/kg b.w.) reduced locomotor activity in mice during the open field test, though its effect was less pronounced than diazepam at 1 mg/kg.

#### 3.5.2. Anticholinesterase Activity

Wetwitayaklung et al. [60] evaluated methanol extracts from various parts of *S. caseolaris* for acetylcholinesterase inhibition. Seed, stamen, meat of fruit, calyx, pneumatophore and leaf extracts exhibited IC_50_ values of 10.52, 55.58, 84.74, 140.12), 126.15, and 146.66 ug/mL, respectively, with the positive control tacrine showing an IC_50_ of 0.01 µg/mL. Additionally, luteolin (**8**) and luteolin glycoside displayed IC_50_ values of 9.31 and 5.87 µg/mL, respectively.

### 3.6. Anticancer Activity

The anticancer potential of *S. caseolaris* has been assessed in vitro using human cancer cell lines, with both crude extracts and isolated compounds evaluated for cytotoxic effects. Tian et al. [22] tested crude methanol extracts from stems and twigs against the SMMC 7721 human hepatoma cell line using an MTT assay. While the crude extract showed weak cytotoxicity, the isolated compound luteolin (**8**) demonstrated potent anticancer activity with an IC_50_ of 2.8 µg/mL. However, this was still less effective than the standard drug mitomycin C, which had an IC_50_ of 1.1 µg/mL. In another study, Wu et al. [34] evaluated nine compounds derived from fruits. Among them, (−)-(R)-nyasol (**34**), (−)-(R)-4′-O-methylnyasol (**35**), and maslinic acid (**43**) showed moderate cytotoxicity against cancer cells, with IC_50_ values ranging from 19.0 to 31.8 µg/mL. These activities were weaker compared to the reference drug 5-fluorouracil, which had an IC_50_ of 5.84 µg/mL.

### 3.7. Metabolic Effects

The metabolic effects of *S. caseolaris* have been studied using a combination of in vivo mouse models to evaluate anti-hyperglycemic, hypocholesterolemic, and anti-obesity activities, and in vitro enzyme inhibition assays, including alpha-amylase and alpha-glucosidase inhibition.

#### 3.7.1. Anti-Hyperglycemic Activity

Tiwari et al. [29] isolated oleanolic acid from the fruit methanol extract, which significantly reduced blood glucose levels by 22%, 21%, and 12% at 30, 60, and 90 min, respectively, after starch administration in mice. Supporting this, Rahmatulla et al. [65] showed that methanol extract dose-dependently reduced blood glucose by up to 41.4% (400 mg/kg b.w.) in an oral glucose tolerance test (OGTT), although the efficacy remained below that of glibenclamide (63.9% at 10 mg/kg b.w.).

Dev et al. [16] further confirmed the antidiabetic efficacy of the ethanol fruit extract (300–500 mg/kg b.w.), which significantly reduced glucose levels in both OGTT and streptozotocin (STZ)-induced diabetic mice. The extract also improved biochemical markers associated with diabetes, with performance comparable to glibenclamide. Similarly, Barman et al. [40] found that leaf ethanol extract (500 mg/kg) significantly reduced glucose levels in alloxan-induced diabetic mice, achieving values comparable to glibenclamide (8.07 vs. 8.05 mmol/L, respectively).

#### 3.7.2. Alpha-Amylase and Alpha-Glucosidase Inhibition

Fang et al. [32] demonstrated that an ellagitannin-rich leaf fraction inhibited α-amylase with an IC_50_ of 7.96 ± 0.05 µg/mL, outperforming acarbose (IC_50_: 24.89 ± 1.49 µg/mL). Tiwari et al. [29] reported that oleanolic acid also inhibited α-glucosidase (IC_50_: 15 µM), while Barman et al. [40] showed moderate α-amylase inhibition by ethanol leaf extract (IC_50_: 37.6 µg/mL), though less potent than acarbose (IC_50_: 16.0 µg/mL).

#### 3.7.3. Hypocholesterolemic Effect

Jariyah et al. [66] demonstrated lipid-lowering activity of *S. caseolaris* fruit water extract in rats. Diets supplemented with 3%, 6%, and 9% extract led to dose-dependent reductions in total cholesterol (32.45–58.87 mg/dL), LDL-c (32.06–55.54 mg/dL), and triglycerides (9.20–23.93 mg/dL), with no significant effect on HDL-c levels.

#### 3.7.4. Anti-Obesity Activity

In a high-fat diet mouse model, Van Thuoc et al. [53] found that hot water extract of the whole fruit of *S. caseolaris* significantly attenuated body weight gain. After five weeks of treatment, extract-fed mice weighed 53.1 g compared to 62.1 g in the untreated high-fat diet control group, indicating promising anti-obesity potential.

### 3.8. Antidiarrheal Activity

In the antidiarrheal efficacy of *S. caseolaris* has been investigated in vivo using castor oil-induced diarrhea models in mice. Ahmed et al. [63] reported that ethanol leaf extract at 500 mg/kg body weight delayed the onset of diarrhea by 1.58 h and reduced defecation frequency, showing comparable effects to standard drug. Bokshi et al. [64] evaluated ethyl acetate stem and chloroform leaf fractions (250–500 mg/kg) and observed prolonged latent periods (0.65–1.40 h) and decreased stool frequency (6–10 stools), relative to the control (0.6 h, 12 stools) and standard drug loperamide (2.35 h, 4 stools).

Fruit extracts also showed notable efficacy. Hosen et al. [49] reported that ethanol-methanol (1:1) extract at 250 mg/kg increased latency to 273 min and reduced defecation by 87.7%, closely matching the effect of loperamide (3 mg/kg). Similarly, Kundu et al. [46] found that ethanol fruit extract (250–500 mg/kg) increased the latent period to 95.8–119 min (versus 32 min control; 171 min loperamide) and decreased defecation by 43.33–64.44%, relative to 76.67% inhibition by loperamide.

### 3.9. Other Activities

#### 3.9.1. Thrombolytic and Coagulation Modulation

Extracts of *S. caseolaris* have shown moderate thrombolytic activity in vitro. Methanol leaf extracts induced clot lysis of 26.05 ± 0.92%, as reported by Chowdhury et al. [67], compared to 63.54 ± 2.61% with the standard thrombolytic agent streptokinase and only 4.2% with distilled water. Similarly, Munira et al. [62] found that methanol extracts of leaf and bark achieved clot lysis rates of 26.27% and 44.67%, respectively, indicating a greater effect from bark-derived compounds (streptokinase: 57.9%; distilled water: 3.62%). In another study, Kundu et al. [46] evaluated the coagulation-modulating activity of fruit ethanol extract. At concentrations of 100, 50, and 25 mg/mL, the extract significantly reduced blood clotting times to 5.016, 5.517, and 5.866 min, respectively, compared to a control value of 6.72 min. Although less effective than phytomenadione (vitamin K1; 3.33 min), these results suggest procoagulant potential of the extract.

#### 3.9.2. Steroid 5a-Reductase Inhibition

The inhibition of steroid 5α-reductase has also been observed. Liu et al. [68] reported that a crude acetone extract and its ethyl acetate and acetone/water soluble fractions inhibited the enzyme with IC_50_ values of 56, 94, and 47 µg/mL, respectively, suggesting potential therapeutic applications in benign prostatic hyperplasia.

#### 3.9.3. Anti-Allergic Activity

Dev et al. [16] evaluated the in vivo anti-allergic activity of *S. caseolaris* fruit ethanol extract using a toluene 2,4-diisocyanate (TDI)-induced allergic model in mice at doses of 300 and 500 mg/kg b.w. Sneezing (26.43 ± 2.59 and 20.33 ± 2.64), scratching (126.71 ± 14.03 and 96.5 ± 10.74), and nasal score (1.2 ± 0.2 and 0.4 ± 0.24) improved compared to untreated TDI-mice (31  ±  2.78; 161.33  ±  6.73; 3) and approached cetirizine (13.75  ±  1.49, 63.0  ±  9.93 and 0.4  ±  0.24). Furthermore, hematological markers were also significantly improved, with results comparable to the standard antihistamine, with a reduction in the total amount of WBC (White blood count) and the number of neutrophils, lymphocytes and eosinophils.

#### 3.9.4. Antipyretic Activity

Using Brewer’s yeast induced pyrexia, Kundu et al. [18] showed in vivo that fruit ethanol extract (250–500 mg/kg b.w.) significantly reduced rectal temperature, though less effectively than ibuprofen (100 mg/kg b.w.).

#### 3.9.5. Anti-Arthritic Activity

Chowdhury et al. [67] evaluated methanol leaf extract in vitro by assessing protein denaturation inhibition. The extract inhibited denaturation by 27.42 ± 0.98% at 31.25 µg/mL and 59.68 ± 1.07% at 1000 µg/mL, compared to diclofenac sodium which inhibited by 52.31 ± 0.56% and 86.67 ± 0.92%, respectively.

#### 3.9.6. Melanin Inhibition

Arung et al. [35] reported in vitro that leaf ethanol extract and luteolin-7-O-glucoside (**12**) inhibited melanin biosynthesis in B16 melanoma cells by up to 46% and 44%, respectively, with low cytotoxicity, suggesting their utility in skin-lightening products.

#### 3.9.7. Anti-Collagenase Activity

Syamsul and Umar [69] demonstrated in vitro that ethanol leaf extracts inhibited collagenase with an IC_50_ of 26.74 ± 0.40 ppm, suggesting potential to prevent collagen degradation and support anti-aging formulations.

## 4. Challenges and Future Perspectives

Research on *S. caseolaris* has notably intensified only over the last decade, with scarce reports before 2010 and a solitary phytochemical analysis by Hogg and Gillan in 1984 [38], underscoring the novelty and promise of this field. To date, investigations remain geographically confined to India, Vietnam, Indonesia, Bangladesh and China, even though it is known that geographical, environmental, and climate factors may influence metabolite profiles in plants [70,71,72]. The focus of previous studies on only a few countries restricts the full understanding of the phytochemistry and bioactivity of *S. caseolaris*. Populations in unstudied regions may experience different environmental pressures, leading to metabolic adaptations that could result in unique secondary metabolites with potentially novel biological activities. Seasonal and geographic chemodiversity, while posing challenges to standardization and reproducibility, also offers unique opportunities for chemical ecology and natural product discovery. Expanding sampling to underexplored regions, particularly those characterized by abiotic stressors such as hypersalinity, high temperatures, or intense solar radiation, could unveil novel stress-induced metabolites, as already been documented in various plant species [73,74,75,76,77,78]. Interestingly, as shown in Table 1, several compounds, including gallic acid, ellagic acid, luteolin, luteolin 7-*O*-glucoside, vanillin, oleanolic acid, lupeol, ursolic acid, sitosterol, cholesterol, and stigmasterol, have been identified across studies from different regions, suggesting the existence of a potential core phytochemical profile conserved across global populations. Future comparative studies employing standardized LC-MS or other advanced analytical protocols would enhance our understanding of *S. caseolaris* chemical ecology and support targeted bioprospecting efforts.

In addition to the geographical limitations of existing studies, while leaves, fruits, bark, stems, and twigs have been studied, no phytochemical investigation of the roots has yet been conducted. This omission is significant, as mangrove roots often accumulate unique metabolites associated with stress adaptation and ecological interaction, many of which hold pharmacological potential [5]. Target exploration of this organ could therefore uncover novel classes of bioactive compounds with distinct therapeutic relevance properties.

The unique adaptability of mangroves for extreme environments has prompted extensive interest in their pharmaceutical potential, particularly for cancer therapy [9]. Mangrove-derived compounds have demonstrated a wide spectrum of anticancer activities in preclinical pharmacological systems [10]. However, while antioxidant and antimicrobial properties of *S. caseolaris* are relatively well documented, studies exploring its anticancer effects remain extremely limited. Only a few investigations have assessed crude extracts or isolated compounds against cancer cell lines, leaving a substantial knowledge gap, especially given the considerable promise shown by other mangrove taxa. Systematic exploration of *S. caseolaris* for anticancer activity should therefore be a high priority and could involve comprehensive cytotoxic screening of crude extracts and fractions on a panel of cancer and normal cell lines to evaluate both efficacy and selectivity, with comparisons to a standard anticancer drug. Fractions demonstrating significant activity would be prioritized for further purification to enhance therapeutic efficacy and selectivity, as well as for mechanistic studies, including apoptosis induction, cell cycle analysis, and molecular pathway investigation, alongside quantification of bioactive compounds to establish structure-activity relationships. Subsequent structural elucidation of active fractions may lead to the identification of novel anticancer candidates suitable for comprehensive in vivo evaluation. This integrated approach would provide a clearer understanding of the anticancer potential of *S. caseolaris* and guide future preclinical and translational research.

Concurrently, *S. caseolaris* populations, like mangroves more broadly, are under significant ecological threat. Coastal development, aquaculture, pollution, and overexploitation have contributed to the degradation of mangrove ecosystems, and the IUCN warns that more than half of global mangrove habitats may collapse by 2050 [79]. Thus, bioprospecting initiatives must be firmly grounded in conservation principles to ensure a reliable, renewable source of plant material. Equally important are frameworks for fair benefit-sharing and engagement with local communities to safeguard ethical and sustainable resource use.

Another persistent limitation lies in the pharmacological validation of *S. caseolaris*. Some bioactivities, such as CNS modulation and metabolic effects, have been explored using both in vitro and in vivo models, but the number of studies is limited, highlighting the need for more comprehensive and systematic evaluations. For several other activities, including anti-inflammatory, analgesic, antidiarrheal, antipyretic, anti-allergic effects, in vivo studies are available but still limited, and further research is necessary to confirm efficacy and reproducibility. In contrast, activities such as antioxidant, antimicrobial, and anticancer effects have been studied in vitro, yet their pharmacological potential has not been validated in vivo systems. Overall, long-term and comprehensive in vivo studies of both extracts and isolated compounds are essential to establish safety, efficacy, and mechanistic insights, thereby providing a solid foundation for eventual clinical evaluations. Moreover, the process of isolating and identifying individual bioactive compounds from the chemically complex matrices of mangrove extracts is often time-consuming and typically requires advanced analytical techniques and substantial quantities of plant material [80], while regulatory pathways for botanical drugs demand rigorous standards of purity, reproducibility and safety. Without dedicated funding, clear intellectual-property frameworks and industry partnerships, progress toward clinical translation will remain slow [81].

Although natural compounds offer several advantages, such as lower toxicity, reduced side effects, and strong therapeutic potential, concerns about their biocompatibility and potential toxicity remain significant obstacles to their development as clinical medicines [82]. As such, innovative drug delivery systems represent a critical direction for future research. Among these, nanotechnology-based approaches are particularly promising. Nanoparticles (NPs) can be engineered to encapsulate or conjugate bioactive molecules, thereby enhancing solubility, stability, and targeted delivery [9,83]. In fact, NPs formulated with mangrove-derived extracts have already demonstrated improved anticancer activity in preclinical models, particularly through mechanisms such as ROS-mediated apoptosis [83,84]. Extending these nanotechnological strategies to *S. caseolaris* could significantly advance its therapeutic application and address current limitations in bioavailability and efficacy.

Thus, realizing the full pharmaceutical value of *S. caseolaris* will require a multifaceted and interdisciplinary approach. Priorities include (1) expanding geographical and seasonal sampling to capture chemodiversity, including populations exposed to extreme environmental conditions that may induce unique metabolites; (2) integrating conservation and sustainable resource management into bioprospecting pipelines; (3) conducting in-depth in vitro and in vivo studies, especially for anticancer potential but also for other pharmacological activities, with the need to increase the number of investigations and emphasis on mechanism-based investigations; (4) exploring under-investigated plant organs, such as roots, which may harbor unique metabolites with distinct pharmacological properties; (5) improving extraction, standardization, and compound isolation techniques; (6) fostering cross-sector funding and research collaborations; and (7) leveraging drug delivery innovations, particularly nanotechnology, to overcome limitations related to bioavailability and targeting. Addressing these interconnected challenges will be crucial to transforming *S. caseolaris* from a largely exploratory subject of study into a practical source of novel bioactive agents with real-world therapeutic applications.

## 5. Materials and Methods

A comprehensive literature review was conducted to gather information on the phytochemistry and pharmacological potential of *S. caseolaris*. Scientific articles and books were retrieved primarily from online databases such as Google Scholar, using combination of keywords including: ‘*Sonneratia*’, ‘*Sonneratia caseolaris*’, ‘phytochemical analysis’, ‘secondary metabolites’, ‘natural products’, ‘bioactive compounds’, ‘biological activities’, ‘antioxidant activity’, ‘cytotoxic activity’, and ‘antimicrobial activity’. In addition, chemical constituents associated with *S. caseolaris* were identified and verified through searches in the CAS SciFinder chemical compound database. All studies and references mentioning *S. caseolaris* were critically assessed for inclusion.

## 6. Conclusions

*S. caseolaris* has emerged as a valuable source of structurally diverse secondary metabolites, including phenolic acids, flavonoids, tannins, terpenoids, steroids, fatty acids and their derivatives, fatty alcohols, aldehydes, and hydrocarbons. These phytochemicals form the basis of a wide array of pharmacological activities demonstrated by extracts prepared using various solvents. Notably, *S. caseolaris* exhibits potent antioxidant and free-radical scavenging properties, largely attributed to its high polyphenolic content. In vivo studies in mice have further revealed significant activity, including central nervous system depressant, antidiarrheal, antihyperglycemic, anti-inflammatory, analgesic, antipyretic, and anti-allergic effects. In addition, although still limited in scope, initial studies suggest anticancer potential, warranting more comprehensive investigation. The plant’s extracts have also demonstrated broad-spectrum antibacterial and antifungal activity, including efficacy against drug-resistant microbial strains. Collectively, these findings support the notion that *S. caseolaris* holds considerable promise as a multi-target therapeutic agent. Its antioxidant and anti-inflammatory properties, in particular, align with its traditional uses for treating infections and inflammatory ailments.

However, current evidence is primarily based on crude extracts and preliminary in vitro studies. A major challenge lies in the variability of extract composition and bioactivity, which can fluctuate depending on factors such as geographical origin, seasonal variation, and, in particular, extraction method. Importantly, no phytochemical analyses of the roots have been reported to date, representing a critical knowledge gap given the ecological and metabolic importance of this organ in mangroves. Future research should therefore prioritize systematic root investigations, alongside the isolation, purification, and structural elucidation of the most bioactive constituents. These compounds should be evaluated not only through mechanistic in vitro studies but also in disease-relevant animal models to assess pharmacological efficacy and safety. In addition, expanding sampling across different geographic regions, integrating standardized extraction and analytical approaches, and applying innovative strategies such as nanotechnology-based delivery systems would provide valuable insights and enhance translational potential. By pursuing these directions, research groups can move beyond exploratory findings and accelerate the development of *S. caseolaris* as a practical source of novel bioactive agents with therapeutic applications.

## Figures and Tables

**Table 1:** Compounds identified in *Sonneratia caseolaris* extracts.

Molecule	Plant Part	Solvent	Method	Region	Ref.
*Phenolic acids and derivatives*					
Gallic acid (**1**)	Leaf	Methanol	LC-MS	India	[31]
			HPLC-DAD	Vietnam	[13]
		Ethanol	LC-MS	India	[31]
			UHPLC-HRMS	Indonesia	[8]
			HPLC-DAD	Vietnam	[13]
		70% aqueous acetone	RP-HPLC	China	[32]
Ellagic acid (**2**)	Fruits	Ethanol	HPLC-DAD	Bangladesh	[16]
	Leaves	70% aqueous acetone	RP-HPLC	China	[32]
Vanillic acid (**3**)	Fruits	Ethanol	HPLC-DAD	Bangladesh	[16]
Chlorogenic acid (**4**)	Leaves	Ethyl acetate	UPLC-ESI-MS/MS	India	[33]
Caffeic acid (**5**)					
p-Coumaric acid (**6**)					
Ferulic acid (**7**)					
*Flavonoids*					
Luteolin (**8**)	Leaves	Methanol	HPLC-DAD	Vietnam	[13]
		Ethanol	MS; 1D- and 2D-NMR	Bangladesh	[21]
	Fruits	Methanol	TLC, IR, ^1^H NMR	India	[29]
		Ethanol	^1^H and ^13^C NMR	China	[34]
	Stem and twigs	Methanol	HR-ESI-MS, ^1^H and ^13^C NMR	China	[22]
Quercetin (**9**)	Leaves	Methanol	LC-MS	India	[31]
		Ethanol	LC-MS	India	[31]
		Ethyl acetate	UPLC-ESI-MS/MS	India	[33]
Apigenin (**10**)	Leaves	Ethyl acetate	UPLC-ESI-MS/MS	India	[33]
Myricetin (**11**)	Fruits	Ethanol	HPLC-DAD	Bangladesh	[16]
	Leaves	Ethyl acetate	UPLC-ESI-MS/MS	India	[33]
Luteolin-7-*O*-glucoside (**12**)	Leaves	Ethanol	MS; 1D- and 2D-NMR	Bangladesh	[21]
			^1^H and ^13^C NMR	Indonesia	[35]
		Methanol	HPLC-DAD	Vietnam	[13]
	Fruits	Ethanol	^1^H and ^13^C NMR	China	[34]
Kaempferol glucoside (**13**)	Bark	Methanol	GC-MS	India	[36]
Quercetin-3-*O*-β-L-arabinopyranoside (**14**)	Stem and twigs	Methanol	HR-ESI-MS, ^1^H and ^13^C NMR	China	[22]
Isovitexin (**15**)	Leaves	Ethanol	UHPLC-HRMS	Indonesia	[8]
Quercitrin (**16**)					
(+)-Dihydrokaempferol (**17**)	Stems and twigs	Methanol	HR-ESI-MS, ^1^H and ^13^C NMR	China	[22]
Riccionidin A (**18**)	Leaves	Ethanol	UHPLC-HRMS	Indonesia	[8]
Cyanidin 3-*O*-[β-D-xylosyl-(1-2)- β-D-galactoside] (**19**)					
Epigallochatechin gallate (**20**)	Leaves	Ethyl acetate	UPLC-ESI-MS/MS	India	[33]
Naringenin (**21**)					
*Tannins*					
Tannic acid (**22**)	Leaves	Ethanol	LC-MS	India	[31]
		Methanol	LC-MS	India	[31]
Methyl gallate (**23**)	Stems and twigs	Methanol	HR-ESI-MS, ^1^H and ^13^C NMR	China	[22]
3,3′-Di-*O*-methyl ether ellagic acid (**24**)					
3,3′,4-*O*-Tri-*O*-methyl ether ellagic acid (**25**)					
*Other phenolic compounds*					
Estragole (**26**)	Fruits	Methanol	GC-MS	Vietnam	[19]
Aspirin (**27**)	Leaves	Ethanol	UHPLC-HRMS	Indonesia	[8]
2-Phenylehtyl (**28**)					
Vanillin (**29**)	Leaves	Ethanol	LC-MS	India	[31]
		Methanol	LC-MS	India	[31]
	Bark	Hexane	GC-TOFMS	Malay Peninsula	[15]
Piperonal (**30**)	Wood and Bark	Hexane	GC-TOFMS	Malay Peninsula	[15]
2,4-Bis(1,1-dimethylethyl)-phenol (**31**)					
3,7,8-Trihydroxy-5,10-dioxo-5,10-dihydrochromeno [5,4,3-cde]chromen-2-olate (**32**)	Fruits	Ethanol	UHPLC-HRMS	Indonesia	[8]
3,5-Di-tert-Butyl-4-hydroxybenzaldehyde (**33**)	Leaves	Ethanol	LC-HRMS	Indonesia	[37]
		Water			
[(–)-(R)-Nyasol (**34**)	Fruits	Ethanol	^1^H and ^13^C NMR	China	[34]
(–)-(R)-4′-*O*-Methylnyasol (**35**)					
3,8-Dihydroxy-6H-benzo[b,d]pyran-6-one (**36**)					
3-Hydroxy-6H-benzo[b,d]pyran-6-one (**37**)					
Benzyl-*O*-β- glucopyranoside (**38**)					
*Terpenoids*					
β-Curcumene (**39**)	Fruits	Methanol	GC-MS	Vietnam	[19]
Cubedol (**40**)					
α-Santonin (**41**)					
Oleanolic acid (**42**)	Fruits	Methanol	TLC, IR, ^1^H NMR	India	[29]
		Ethanol	^1^H and ^13^C NMR	China	[34]
	Stems and twigs	Methanol	HR-ESI-MS, ^1^H and ^13^C NMR	China	[22]
	Bark	Methanol	GC-MS	India	[36]
Maslinic acid (**43**)	Fruits	Ethanol	^1^H and ^13^C NMR	China	[34]
Lupeol (**44**)	Stems and twigs	Methanol	HR-ESI-MS, ^1^H and ^13^C NMR	China	[22]
	Bark	Methanol	GC-MS	India	[36]
Ursolic acid (**45**)	Stems and twigs	Methanol	HR-ESI-MS, ^1^H and ^13^C NMR	China	[22]
	Bark	Methanol	GC-MS	India	[36]
Abietin (**46**)	Leaves	Ethanol	UHPLC-HRMS	Indonesia	[8]
Squalene (**47**)	Leaves	Chloroform-methanol (1:1)	GC	Australia	[38]
Rhodopin (**48**)	Fruits	Methanol	GC-MS	Vietnam	[19]
Lup-20(29)-en-3β,24-diol (**49**)	Stems and twigs	Methanol	HR-ESI-MS, ^1^H and ^13^C NMR	China	[22]
3β-*O*-(E)-Cumaroyl-alphitolinsaeure (**50**)					
3β-Acetyl-oleanolic acid (**51**)					
3β,13β-Dihydroxy-urs-11-en-28-oic acid-13-lactone (**52**)					
3β-Hydroxy-20(29)-lupen-24-oic acid (**53**)					
Betulin (**54**)					
1H-Cycloprop[e]azulen-4-ol, decahydro-1,1,4,7-tetramethyl-, [1ar (1aà,4á,4aá,7à,7aá,7bà)]- (**55**)	Wood	Hexane	GC-TOFMS	Malay peninsula	[15]
*Steroids*					
Campesterol (**56**)	Leaves	Chloroform-methanol (1:1)	GC	Australia	[38]
28-Isofucosterol (**57**)					
Sitosterol (**58**)					
	Bark	Methanol	GC-MS	India	[36]
	Stem and twigs	Methanol	HR-ESI-MS, ^1^H and ^13^C NMR	China	[22]
Cholesterol (**59**)	Leaves	Chloroform-methanol (1:1)	GC	Australia	[38]
	Stem and twigs	Methanol	HR-ESI-MS, ^1^H and ^13^C NMR	China	[22]
Stigmasterol (**60**)	Leaves	Chloroform-methanol (1:1)	GC	Australia	[38]
	Stem and twigs	Methanol	HR-ESI-MS, ^1^H and ^13^C NMR	China	[22]
	Leaves	Acetone	UV-Vis, FTIR, ^1^H and ^13^C NMR, 2D-NMR	Indonesia	[39]
Cholest-5-ene-diol (**61**)	Bark	Methanol	GC-MS	India	[36]
	Stem and twigs	Methanol	HR-ESI-MS, ^1^H and ^13^C NMR	China	[22]
β-Sistosterol-β-D-glucopyranoside (**62**)	Fruits	Methanol	TLC, IR, ^1^H NMR	India	[29]
Prednisone (**63**)	Fruits	Methanol	GC-MS	Vietnam	[19]
β-Sitosterol palmitate (**64**)	Stem and twigs	Methanol	HR-ESI-MS, ^1^H and ^13^C NMR	China	[22]
Stigmast-5-en-3β-O-(6-O-hexadecanoyl-β-D-glucopyranoside) (**65**)					
Daucosterol (**66**)					
6′-*O*-Acetyl-β-daucosterol (**67**)					
*Fatty acid and derivatives*					
13S-Hydroxyoctadecadienoic acid (**68**)	Leaves	Ethanol	UHPLC-HRMS	Indonesia	[8]
9-Hydroperoxy-11-(3-pentyl-2-oxiranyl)-10-undecenoate (**69**)					
9,12,13-Trihydroxy-10-octadecenoate (**70**)					
Octanoid acid (**71**)	Bark	Hexane	GC-TOFMS	Malay peninsula	[15]
Butanoic acid (**72**)	Leaves	Methanol	GC-MS	Vietnam	[19]
Dodecanamide (**73**)	Wood and Bark	Hexane	GC-TOFMS	Malay peninsula	[15]
Myristynoyl pantetheine (**74**)	Leaves	Methanol	GC-MS	Vietnam	[19]
*Fatty aldehydes*					
2-Heptenal (**75**)	Wood and bark	Hexane	GC-TOFMS	Malay peninsula	[15]
2-Octenal (**76**)					
Nonanal (**77**)					
2,4-Decadienal (**78**)					
2-Undecenal (**79**)					
Hexadecanal (**80**)					
Tetradecanal (**81**)	Wood				
Octadecanal (**82**)	Bark				
2-Nonenal (**83**)					
Decanal (**84**)					
Tridecanedial (**85**)	Leaves	Methanol	GC-MS	Vietnam	[19]
*Fatty alcohols*					
13-Heptadecyn-1-ol (**86**)	Leaves	Methanol	GC-MS	Vietnam	[19]
2-Hexadecanol (**87**)					
1-Octanol (**88**)					
Falcarinol (**89**)					
Trans-9-hexadecen-1-ol (**90**)	Wood	Hexane	GC-TOFMS	Malay peninsula	[15]
*Other lipid-derived compounds*					
4,8,12,16-Tetramethylheptadecan-4-olide (**91**)	Wood	Hexane	GC-TOFMS	Malay peninsula	[15]
Oxacycloheptadec-8-en-2-one (**92**)					
13-Methyl-oxacyclotetradecane-2,11-dione (**93**)					
Tert-hexadecanethiol (**94**)	Leaves	Methanol	GC-MS	Vietnam	[19]
Triacetin (**95**)					
Azelaic acid (**96**)	Leaves	Ethanol	UHPLC-HRMS	Indonesia	[8]
*Hydrocarbons*					
Pentadecane (**97**)	Wood and Bark	Hexane	GC-TOFMS	Malay peninsula	[15]
1-Hexadecene (**98**)					
1-Docosene (**99**)					
Octacosane (**100**)					
Hentriacontane (**101**)					
Heptadecane (**102**)	Wood				
2-Methyl-nonadecane (**103**)					
Octadecane (**104**)					
Tetracosane (**105**)					
Heptacosane (**106**)					
Eicosane (**107**)	Bark				
17-Pentatriacontene (**108**)					
Isobutane (**109**)					
3-Methyl-hexane (**110**)					
1-Chloro-heptacosane (**111**)					
*Polysaccharides*					
Hexose (**112**)	Leaves	Ethanol	UHPLC-HRMS	Indonesia	[8]
Sorbitol (**113**)					
Rhamnose (**114**)		Water (low-molecular-weight polysaccharide fraction)	RP-HPLC	India	[30]
Xylose (**115**)					
Mannose (**116**)					
Galactose (**117**)					
*Other compounds*					
Diisobutyl phthalate (**118**)	Leaves	Water	LC-HRMS	Indonesia	[37]
Bis(3,5,5 trimethylhexyl)phthalate (**119**)					
Monobutyl phthalate (**120**)					
Bis(2 ethylhexyl)phthalate (**121**)					
Betaine (**122**)					
Choline (**123**)					
Hexamethylenetetramine (**124**)					
2,2,6,6 tetramethyl 1 piperidinol (TEMPO) (**125**)					
Caprolactam (**126**)					
2-[(2-chlorobenzyl)sulfanyl]-4,6-dimethylnicotinonitrile (**127**)					
Zearalenone (**128**)					
Tributyl phosphate (**129**)					
Bis(4-ethylbenzylidene)sorbitol (**130**)					
DL-arginine (**131**)					
Bis(2-ethylhexyl)benzene-1,2-dicarboxylate (**132**)	Stems and twigs	Methanol	HR-ESI-MS, ^1^H and ^13^C NMR	China	[22]
Safrole (**133**)	Fruits	Methanol	GC-MS	Vietnam	[19]
1,2-Benzenedicarboxylic acid, bis(2-methylpropyl) ester (**134**)	Wood and bark	Hexane	GC-TOFMS	Malay peninsula	[15]
7,9-Di-tert-butyl-1-oxaspiro(4,5)deca-6,9-diene-2,8-dione (**135**)					
Ethaneperoxoic acid, 1-cyano-1-[2-(2-phenyl-1,3-dioxolan-2-yl)ethyl]pentyl esterv (**136**)					
Trimethylamine (**137**)	Wood				
1,2-Benzenedicarboxylic acid, diisooctyl ester (**138**)					
Propiolactone (**139**)	Bark				
Diethyl phthalate (**140**)					
1,2-Benzenedicarboxylic acid, mono(2-ethylhexyl) ester (**141**)					

**Table 2:** Total phenolic content (TPC) and total flavonoid content (TFC) of *Sonneratia caseolaris*.

Plant part	Solvent	TPC (mg GAE/g)	TFC (mg QE/g)	Ref.
Leaves	Methanol	200	-	[13]
		1.52 ± 0.02	1.98 ± 0.08	[37]
	Ethanol	182.89 ± 1.76	22.70 ± 0.48	[8]
		50.03	-	[41]
		219.53	454.88	[40]
	Ethanol 70%	74.77	-	[41]
	Ethyl acetate	5.83	-	
	n-Hexane	4.67	-	
	Water	0.23–1.00	0.55–0.96	[37]
Fruits	Ethanol	122	613	[18]
		12.21 ± 1.31	26.06 ± 0.30	[42]
	Methanol	82.27 ± 0.41	41.0 ± 0.34	[19]
	Methanol (n-butanol fraction)	82.67 ± 0.81	9.13 ± 0.34	
	Methanol (ethyl acetate fraction)	77.67 ± 0.32	26.28 ± 0.93	
	Methanol (aqueous fraction)	70.26 ± 0.35	1.81 ± 0.24	
	Methanol (Hexane fraction)	59.58 ± 2.70	16.54 ± 0.44	
Bark	Ethanol	63.00	-	[43]
		50.70 ± 0.74	90.04 ± 3.57	[36]
	Ethyl acetate	60.25	-	[43]
	Chloroform	36.25	-	
	Petroleum ether	26.28	-	

**Table 3:** Antimicrobial activities of *Sonneratia caseolaris* extracts.

Plant Part	Solvent	Conc. Tested	Bacterial/Fungal Species	Activity	Results	Standard	Ref.
Leaves (L), roots (E), flower [stamen (T) and calyx (P)], and fruit [meat of fruit (F), persistent calyx of fruit (C), and seeds (S)]	Methanol	-	AS *S. aureus* (+)	MIC (mg/mL)	0.2 (L), 0.4 (E, S, P, T, F, C)	0.0062 (CHL)	[50]
			AS *B. subtilis* (+)		0.2 (L, E), 0.4 (S, P, T, F, C)	0.0015 (CHL)	
			AS *B. megaterium* (+)		0.2 (L, E, S, P), 0.4 (T, F, C)	0.0015 (CHL)	
			AS *E. coli* (−)		0.2 (L, E, S, P, T, F, C)	0.0062 (CHL)	
			AS *P. aeruginosa* (–)		0.2 (L, E, S, P, T, F, C)	0.025 (CHL)	
			AR *S. aureus* (+)		0.4 (L, E, S, P, T, F, C)	0.0062 (CHL)	
			AR *E. faecalis* (+)		0.2 (L, S), 0.4 (E, P, T, F, C)	0.0062 (CHL)	
			AR *E. faecium* (+)		0.2 (L, E, S, P), 0.4 (T, F, C)	0.0062 (CHL)	
			AR *E. coli* (−)		0.2 (L, E, S, P, T, F, C)	0.0062 (CHL)	
			AR *P. aeruginosa* (−)		0.4 (L, E, S, P, T, F, C)	0.1 (CHL)	
			*AR A. baumannii* (−)		0.2 (P, T, C), 0.4 (L, E, S, F)	n/a (CHL)	
Leaves	Ethanol (E), Water (W)	2 mg/disc (CHL 20 μg/disc; AMB 40 μg/disc)	*S. aureus* (+)	ZOI (mm); MIC (µg/mL)	n/a; n/a (E), n/a; n/a (W)	30; 7.81 (CHL)	[51]
			*B. subtilis* (+)		n/a; n/a (E), n/a; n/a (W)	30; 21.3 (CHL)	
			*M. luteus* (+)		n/a; n/a (E), n/a; n/a (W)	24; 31.3 (CHL)	
			*E. coli* (−)		15; 500 (E), 13; 250 (W)	30; 31.3 (CHL)	
			*P. aeruginosa* (−)		n/a; n/a (E), n/a; n/a (W)	15; 125 (CHL)	
			*C. albicans*		n/a; n/a (E), 15; 125 (W)	17; 250 (AMB)	
Seeds	Methanol	-(AMP 10 μg/disc)	*S. aureus* (+)	ZOI (mm)	12–14	29–35 (AMP)	[52]
			*E. coli* (−)		n/a	0–10 (AMP)	
			*C. albicans*		17–18	n/a (AMP)	
Leaves	Water (W), Methanol (M)	10 µg/ml	*Vibrio* spp. (−)	ZOI (mm); MIC (µg/mL)	n/a; 1.6–6.3 (W), n/a-18.3; 0.1–0.2 (M)	-	[47]
Bark	Hexane (H), Benzene (B), Chloroform (C), Mthanol (M), Water (W)	1.5 mg/disc (AMP 3 µL/disc 125 µg/mL; CHL 3 µL/disc 10 mg/mL; FLC 3 µL/disc 10 mg/Ml)	*B. subtilis* (+)	ZOI (mm); MIC (mg/mL)	9.33 ± 0.58;-(H), 8.33 ± 0.58;-(B), 7.17 ± 0.29;-(C), 18.33 ± 0.76; 3.90 (M), 15.83 ± 0.29; 15.62 (W)	20.83 ± 0.76;-(AMP)	[36]
			*B. coagulans* (+)		9.17 ± 0.29;-(H), 9.50 ± 0.50;-(B), 10.17 ± 0.29; 7.81 (C), 19.50 ± 0.50; 7.81 (M), n/a;-(W)	22.50 ± 0.50;-(AMP)	
			*E. coli* (−)		n/a;-(H, B, C, M, A)	13.00 ± 1.04;-(AMP)	
			*S. cerevisiae* (+)		n/a;-(H, B, C, M, A)	20.00 ± 1.73;-(FLC)	
			*P. vulgaris* (−)		n/a;-(H, B, C), 12.67 ± 0.58; 62.50 (M), 8.67 ± 0.58; 125 (W)	17.17 ± 1.26;-(CHL)	
Fruits	Water	-	*E coli* (−)	ZOI (mm)	18–21	-	[53]
			*V. cholerae* (−)		32–35	-	
			*S. typhimurium* (−)		25–27	-	
			*B. subtilis* (+)		24–29	-	
Fruits	Methanol	60–90% (CHL 0.025%)	*S. aureus* (+)	ZOI (mm)	8.81 (80%)	12.73 (CHL)	[54]
		5–20% (CHL 0.025%)	*E. coli* (−)		7.17 (15%)	11.53 (CHL)	
		20–35% (MTZ 0.15%)	*C. albicans*		7.03 (30%)	9.13 (MTZ)	
Fruits	Ethyl acetate (EA), Ethanol 70% (E)	2.5–15% (STM 10 µg/disc)	*E. coli* (−)	ZOI (mm)	14.50 (EA 15%), 13.15 (E 12.5%)	27.61 (STM)	[55]
			*S. aureus* (+)		7.46 (EA 15%), 13.37 (E 10%)	27.61 (STM)	
			*C. albicans*		11.67 (EA 15%), 28.50 (E 10%)	31.40	
Stems and leaves	Methanol: ethyl acetate (EAFS, EAFL), chloroform (CFS, CFL), carbon tetrachloride (CTFS, CAFL) fractions	500 µg/disc (CIP 5 µg/mL)	*E. coli* (−)	ZOI (mm)	0 (EAFS), 7.5 ± 0.08 (EAFL), 7.5 ± 0.09 (CFS), 7.5 ± 0.13 (CFL), 5.5 ± 0.07 (CTFS), 0 (CTFL)	11.5 ± 0.07 (CIP)	[48]
			*S.aureus* (+)		6 ± 0.11 (EAFS), 7.5 ± 0.11 (EAFL), 7.5 ± 0.09 (CFS), 8 ± 0.09 (CFL), 8.5 ± 0.13 (CTFS), 0 (CTFL)	11 ± 0.15 (CIP)	
			*S. dysenteriae* (−)		6.5 ± 0.07 (EAFS), 8.5 ± 0.06 (EAFL), 7.5 ± 0.16 (CFS), 5.5 ± 0.20 (CFL), 7 ± 0.09 (CTFS), 0 (CTFL)	13.5 ± 0.05 (CIP)	
			*S. typhi* (−)		8.5 ± 0.11 (EAFS), 8.5 ± 0.12 (EAFL), 5.5 ± 0.06 (CFS), 7.5 ± 0.13 (CFL), 7 ± 0.13 (CTFS), 0 (CTFL)	12 ± 0.09 (CIP)	
			*S. paratyphi* (−)		11.5 ± 0.1 (EAFS), 6.5 ± 0.17 (EAFL), 6 ± 0.07 (CFS), 5.5 ± 0.07 (CFL), 9 ± 0.09 (CTFS), 0 (CTFL)	15 ± 0.15 (CIP)	
Levaes	Ethyl acetate	0.05–50 mg/mL	*C. albicans*	MIC (mg/mL)	4	0.5 (AMB)	[33]
			*C. tropicalis*		16	0.5 (AMB)	
			*C. auris*		32	1 (AMB)	
Fruits	Ethanol-methanol (1:1)	2 mg/disc	*E. coli* (−)	ZOI (mm)	16.7	-	[49]
			*Klebsiella* sp. (−)		17.0	-	
			*S. boydii* (−)		14.7	-	
			*S. sonnei* (−)		15.7	-	
			*S. aureus* (+)		15.7	-	
Leaves	Ethanol	20,000 ppm (VAN 5 μg/disc)	Methicillin-resistant *S. aureus (+)*	ZOI (mm)	11.92	24 (VAN)	[8]
Leaves	n-Hexane (H), Ethyl acetate (EA), Ethanol 70% (E70), Ethanol 90% (E90)	2.5–15%; (STM 5 μg/disc)	*S. aureus* (+)	ZOI (mm)	3.73 (H 10%), n/a (EA 12.5%), 6.98 (E70 10%), n/a (E90 10%)	41.7–44.54 (STM)	[41]
			*E. coli* (−)		1.82 (N 10%), 1.03 (EA 10%), 1.57 (E70 15%), 2.18 (E90 15%)	28.28–33.50 (STM)	
			*C. albicane*		5.70 (N 15%), n/a (EA 15%), 5.00 (E70 12.5%), n/a (E90 15%)	16.90–24.00	
Leaves	Water (low-molecular-weight polysaccharide fraction)	20 mg/mL	*S. pneumoniae* (+)	Antibiofilm activity (%)	78.72 ± 1.99	-	[30]
			*P. aeruginosa* (−)		65.89 ± 1.68	-	
			*S. aureus* (+)		52.04 ± 1.35	-	
			*E. coli* (–)		49.54 ± 0.79	-	
			*S. typhi* (−)		40.17 ± 1.53	-	
Fruit	Methanol extract (M): hexane (HF), ethyl acetate (EAF), n-butanol (BF), and aqueous (WF) fractions	50 mg/mL	*E.coli* (−)	ZOI (mm)	21.00 ± 1.00 (M), 14.67 ± 0.58 (HF), 13.67 ± 0.58 (AEF), 17.67 ± 0.47 (BF), 17.33 ± 1.53 (AF)	-	[19]
			*S. aureus* (+)		21.00 ± 1.00 (M), 12.67 ± 0.58 (HF), 14.67 ± 0.58 (AEF), 11.33 ± 1.15 (BF), 16.33 ± 0.58 (AF)	-	
			*B. subtilis* (+)		18.67 ± 0.58 (M), 16.33 ± 0.58 (HF), 17.33 ± 0.58 (AEF), 18.33 ± 0.58 (BF), 16.67 ± 0.58 (AF)	-	
Leaves	Ethanol	-	*E. coli* (−)	ZOI (mm); MIC (mg/mL)	26; 0.06	-	[13]
			*S. aureus* (+)		33; 0.06	-	
			*S. typhimurium* (−)		25; 0.04	-	

n/a: no inhibitory activity detected; AR = antibiotic resistant, AS = antibiotic sensitive; CHL = chloramphenicol, AMB = amphotericin, AMP = ampicillin, FLC = fluconazole, MTZ = metronidazole, STM = streptomycin, CIP = ciprofloxacin, VAN = vancomycin.

**Table 4:** DPPH scavenging activity of *Sonneratia caseolaris* extracts.

Plant Part	Solvent	Fraction	Results: IC_50_ (or %)	Standard: IC_50_ (or %)	Ref.
Leaves	Ethanol	-	1.92 ± 0.38 μg/ml	AA: 12 ± 1.29 μg/ml	[51]
			4.25 ppm	AA: 5.25 ppm	[8]
			23.84 μg/mL	-	[13]
			26.30 μg/mL	AA: 3.16 μg/mL	[57]
			27.0 µg/mL	AA: 12.0 µg/mL	[40]
			171 ppm	AA: 20.5 ppm	[39]
			80.21% at 2.5 mg/mL	-	[41]
		Ethyl acetate	12.0 ± 0.12 µg/mL	AA: 8.0 ± 0.12 µg/mL	[48]
		chloroform	19.0 ± 0.07 µg/mL		
		Carbon tetrachloride	49.0 ± 0.05 µg/mL		
	Ethanol 70%	-	75.89% at 2.5 mg/mL	-	[41]
	Ethyl acetate	-	16.71% at 2.5 mg/mL		
	n-Hexane	-	22% at 2.5 mg/mL		
	Acetone	-	166.2 ppm	-	[39]
	Water	-	89 mg/mL	-	[37]
	70% aqueous acetone	Ellagitannin-rich fraction	69.39 ± 0.29 µg/mL	BHA: 116.52 ± 0.95 µg/mL	[32]
	Water	Low-molecular-weight polysaccharide fraction	41.33 ± 0.82% at 3.2 mg/ml	AA: 85.26 ± 0.96% at 3.2 mg/mL	[30]
Stems	Ethanol	Ethyl acetate	138 ± 0.8 µg/mL	AA: 8.0 ± 0.12 µg/mL	[48]
		Chloroform	69.0 ± 0.21 µg/mL		
		Carbon tetrachloride	201.0 ± 0.15 µg/mL		
Fruits	Ethanol	-	1.16 ± 0.76 µg/mL	AA: 5.28 ± 0.58 µg/mL	[42]
		-	87 μg/ml	AA: 15 μg/ml	[18]
		n-Hexane	191.31 ppm	AA: 3.70 ppm	
		Ethyl acetate	96.02 ppm		
		Butanol	371.16 ppm		
	Ethanol-methanol (1:1)	-	48.1% at 50 µg/mL	-	[58]
	Water	-	7.3 mg/mL	-	[53]
Bark	Ethanol	-	4.57 µg/mL	BHT: 3.25 µg/mL	[43]
	Petroleum ether	-	12.32 µg/mL		
	Ethyl acetate	-	13.09 µg/mL		
	Chloroform		192.27 µg/mL		
	Methanol		21.74 µg/mL	Quercetin: 10.14 µg/mL.	[36]

AA = ascorbic acid, BHA = butylated hydroxyanisole, BHT = butylated hydroxytoluene.
